# Comparative analysis of adverse event risks in breast cancer patients receiving pembrolizumab combined with paclitaxel *versus* paclitaxel monotherapy: insights from the FAERS database

**DOI:** 10.3389/fphar.2024.1345671

**Published:** 2024-08-21

**Authors:** Yilun Li, Xiaolu Yang, Li Ma

**Affiliations:** ^1^ Department of Breast Disease Center, the Fourth Hospital of Hebei Medical University, Shijiazhuang, China; ^2^ Hebei Medical University, Shijiazhuang, China; ^3^ Hebei Key Laboratory of Breast Cancer Molecular Medicine, Shijiazhuang, China

**Keywords:** pembrolizumab, paclitaxel, breast cancer, adverse event, FAERS

## Abstract

**Objective:**

This study aimed to evaluate the risk of adverse events (AEs) in breast cancer patients treated with pembrolizumab combined with paclitaxel *versus* those receiving pembrolizumab or paclitaxel monotherapy, using the FDA Adverse Event Reporting System (FAERS) database.

**Methods:**

Data were extracted from the FAERS database for breast cancer patients treated with pembrolizumab combined with paclitaxel or with pembrolizumab or paclitaxel monotherapy from Q1 2016 to Q2 2023. Disproportionation analysis was performed by calculating the reporting odds ratio (ROR) with corresponding 95% confidence interval (95% CI), the information component (IC), and the lower bound of the information component 95% confidence interval (IC025) to identify potential safety signals.

**Results:**

No significant difference in AEs was observed between the combined treatment group and the pembrolizumab monotherapy group. However, the combined treatment group exhibited a substantial increase in AE risk compared to the paclitaxel monotherapy group. The most significant increases in AE risk were adrenal insufficiency (ROR = 189.94, 95% CI 25.41–1419.7, IC = 3.37, IC025 = 1.59), hypophysitis (ROR = 99.46, 95% CI 12.72–777.4, IC = 3.31, IC025 = 1.44), and myocarditis (ROR = 69.5, 95% CI 8.55–565.23, IC = 3.25, IC025 = 1.33). The time-to-event for combined treatment was 35 (34–70) days, for pembrolizumab was 43 (35–90) days, and for paclitaxel was 42 (37–76) days. The combination therapy group demonstrated significantly shorter intervals to the onset of adrenal insufficiency (*p* = 0.008), myocarditis (*p* < 0.001), and immune-related enterocolitis (*p* = 0.009).

**Conclusion:**

Analysis of the FAERS database indicates that combination therapy significantly elevates the risk of adrenal insufficiency, myocarditis, hypophysitis, and immune-related enterocolitis compared to paclitaxel monotherapy. These findings provide critical insights for clinicians in predicting and managing potential AEs associated with this treatment regimen.

## Introduction

Breast cancer is one of the most prevalent cancers in women and ranks as the second leading cause of cancer-related deaths, following lung cancer (Giaquinto et al., 2022). This highlights the critical public health threat posed by breast cancer and the pressing need for effective prevention and treatment strategies.

The disease is classified into distinct subtypes based on the expression of estrogen receptor (ER), progesterone receptor (PR), and human epidermal growth factor receptor-2 (HER2): Luminal A, Luminal B, HER2, and triple-negative breast cancer (TNBC) (Goldhirsch et al., 2013). This classification facilitates tailored treatments and improves prognosis for breast cancer patients. Among these subtypes, TNBC is particularly aggressive, marked by the absence of ER and PR, and lack of HER2 amplification or overexpression (Cortes et al., 2022). This receptor deficiency renders endocrine and HER2-targeted therapies ineffective, making cytotoxic chemotherapy the standard treatment (Gucalp and Traina, 2011). However, overall survival and response duration remain limited, underscoring the need for novel treatment strategies to enhance outcomes.

Pembrolizumab, an anti-programmed death-ligand 1 (PD-L1) agent, has shown sustained antitumor activity in advanced TNBC, particularly as a first-line therapy (Adams et al., 2019a; Adams et al., 2019b; Winer et al., 2021). Enhanced clinical responses were noted in patients with high PD-L1 expression. The Phase 3 KEYNOTE-355 trial evaluated whether adding pembrolizumab could enhance the antitumor efficacy of chemotherapy, including paclitaxel and non-paclitaxel platinum-based regimens, for previously untreated locally recurrent inoperable or metastatic TNBC. Preliminary data from a pre-specified interim analysis indicated that in patients with a combined PD-L1 positive score (CPS) ≥10, pembrolizumab combined with chemotherapy significantly prolonged progression-free survival compared to placebo plus chemotherapy in PD-L1-stained cells with CPS (Cortes et al., 2020).

Regarding adverse events (AEs) associated with combination therapy, common occurrences included anemia (49.1% in the pembrolizumab chemotherapy group vs. 45.9% in the placebo chemotherapy group), neutropenia (41.1% vs. 38.1%), and nausea (39.3% vs. 41.3%). Pneumonia and kidney injury were significant contributors to AEs leading to death (Cortes et al., 2022). Pembrolizumab-induced AEs affected 54 (18%) of 294 patients in the pembrolizumab group and 21 (8%) of 276 patients in the paclitaxel group (Winer et al., 2021). In the pembrolizumab group, grade 3–5 serious AEs occurring in two or more patients included hepatitis, hypophysitis, and pneumonia (Shitara et al., 2018). The substantial impact of immunotherapy combined with chemotherapy on patient health necessitates identifying potential adverse reactions to ensure the safety of combination therapy.

The U.S. Food and Drug Administration (FDA) Adverse Event Reporting System (FAERS) is a publicly accessible database containing voluntary AE reports from healthcare professionals, consumers, and manufacturers. Its primary objective is to support the FDA’s post-market safety surveillance of drugs and biologics. Leveraging FAERS data for AE database mining provides an effective method to identify associations between drugs and AEs. The extensive and regularly updated FAERS knowledge base allows adverse reaction database mining to more accurately mirror real-world research trends. Recently, large spontaneous AE reporting system databases have gained prominence in pharmacovigilance studies for drug-safety assessment. Currently, no data mining studies based on the FAERS database address AEs in breast cancer patients treated with pembrolizumab in combination with paclitaxel (Chen et al., 2023).

This study investigated the differential risk of AEs in breast cancer patients treated with pembrolizumab combined with paclitaxel *versus* those treated with pembrolizumab or paclitaxel alone, utilizing the FAERS database. Additionally, the median onset time for AEs across different groups was analyzed. This research provides critical insights for improving the prevention and management of AEs in combination therapy for breast cancer.

## Methods

### Data sources and preprocessing

This study analyzed adverse reactions in patients treated with pembrolizumab and paclitaxel using data from the FAERS. Data were sourced from the FAERS database, specifically extracting AE reports from the FDA’s website (http://www.fda.gov/Drugs/InformationOnDrugs/ucm135151HTM). The FAERS database aggregates spontaneous AE reports from healthcare professionals, manufacturers, and consumers worldwide. Datasets on Patient Demographic and Management Information (DMEO), Drug and Biological information (DRUG), Adverse Event (REAC, AEs), and Patient Outcome (OUTC) were employed for analysis. These datasets were integrated using unique identifiers assigned to each FAERS report. AEs were classified based on the Medical Dictionary of Regulatory Activities (MedDRA, http://www.meddra.org/), 1st edition, Version 20.0, and coded using MedDRA^®^ Preferred Terms (PT).

Drug data inclusion criteria are specified as follows: 1. For the Drug A (pembrolizumab) group: inclusion criteria encompass suspected drugs containing Drug A (Primary Suspect Drug, PS), with Drug B excluded from the drug combination (Secondary Suspect Drug, SS; Concomitant, C; Interacting, I). 2. For the Drug B (paclitaxel) group: inclusion criteria require suspected drugs containing Drug B (PS), with the drug combination excluding Drug A (SS, C, I). 3. For the Drug A+ Drug B group: inclusion criteria cover suspected drugs containing both Drug A (PS) + Drug B (SS, C, I) or Drug B (PS) + Drug A (SS, C, I).

A search for drugs related to pembrolizumab and paclitaxel was conducted using “pembrolizumab’ and ‘PACLITAXEL’ as keywords, respectively. Concurrently, the terms ‘breast cancer’, ‘advanced breast cancer’, ‘breast cancer female’, ‘breast cancer female nos’, ‘breast cancer *in situ*’, ‘breast cancer male’, ‘breast cancer metastatic’, ‘breast cancer nos’, ‘breast cancer recurrent’, ‘breast cancer stage i′, ‘breast cancer stage ii’, ‘breast cancer stage iii’, ‘breast cancer stage iv’, ‘estrogen receptor positive breast cancer’, ‘her2 negative breast cancer’, ‘her2 positive breast cancer’, ‘her-2 positive breast cancer’, ‘hormone receptor negative her2 positive breast cancer’, ‘hormone receptor positive breast cancer’, ‘hormone receptor positive her2 negative breast cancer’, ‘hormone refractory breast cancer’, ‘infiltrating ductal breast cancer’, ‘inflammatory breast cancer’, ‘invasive ductal breast cancer’, ‘metastatic breast cancer’, ‘node-negative breast cancer’, ‘node-positive breast cancer’, ‘oestrogen receptor positive breast cancer’, ‘triple negative breast cancer’, and ‘triple positive breast cancer’ were utilized as search terms for breast cancer.

**FIGURE 1:**
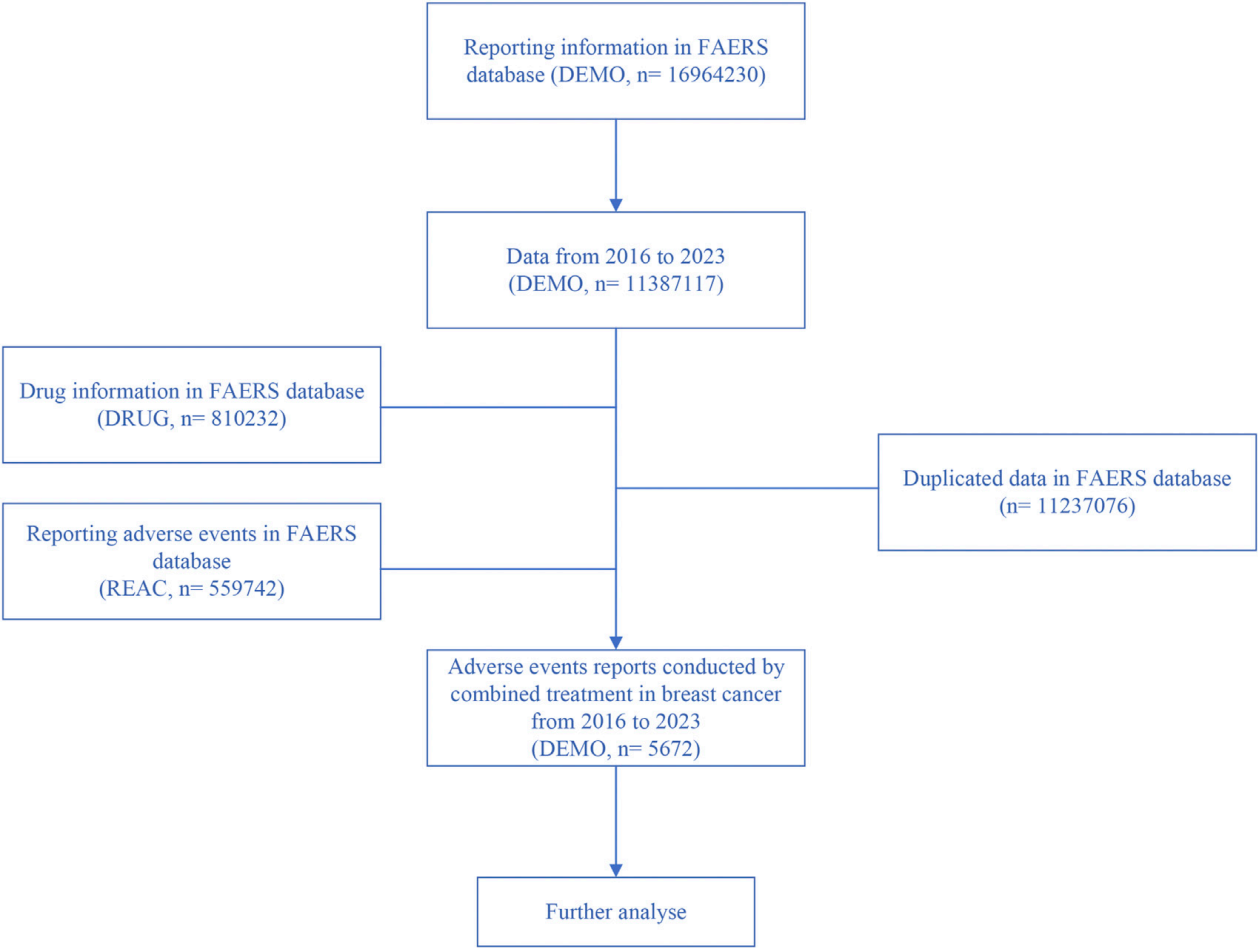
Flowchart illustrating the screening process for adverse reactions based on inclusion criteria.

The dataset spans from Q1 2016 to Q2 2023. After excluding duplicate reports with identical case numbers, 5,672 reports were retained for analysis, as shown in Figure 1.

### Statistical analysis

Given the limitations of the spontaneous reporting system in calculating AE rates, disproportionality analysis was utilized. This method estimates the expected reporting frequency from all drugs and events in the database, identifying drug-related AEs as signals reported more frequently than anticipated. This approach has also been used to evaluate the safety of anti-PD-1/PD-L1 therapy (Almenoff et al., 2007). The reported odds ratio (ROR), 95% confidence interval (95% CI), and the lower bound of the two-way 95% interval of the information components (IC025) were computed (Bate et al., 2002; Bate and Evans, 2009). The formulas for calculating ROR and 95% CI were as follows:

$$ROR=\frac{ad}{bc}$$

$$95\%CI=e^{\ln\left(ROR\right)\pm 1.96SQRT\left(\frac{1}{a}+\frac{1}{b}+\frac{1}{c}+\frac{1}{d}\right)}$$

Here, ‘a’ denoted the number of patients experiencing adverse reactions in the combination therapy group, ‘b’ represented those in the non-combination therapy group, ‘c’ corresponded to patients receiving paclitaxel/pembrolizumab therapy, and ‘d’ pertained to those not receiving paclitaxel/pembrolizumab therapy. A significant safety signal was identified when ROR > 1, and IC and IC025 > 0 (Chen et al., 2022). The timing of AEs was calculated as Time of AE = Date of event - Start date of treatment. Median and interquartile ranges (IQR) were determined to represent time to onset. All statistical analyses and graphical representations were conducted using R Studio (version 4.1.2; Boston, MA, United States).

## Results

### Clinical baseline characteristics and adverse event trends

**FIGURE 2:**
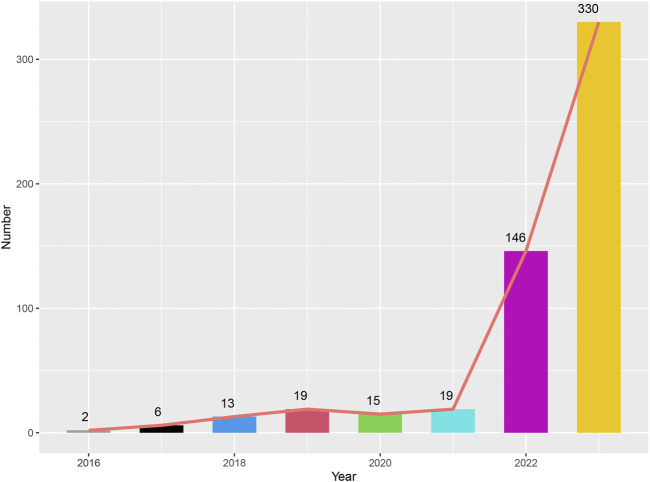
Graph depicting the number of adverse events reported in patients treated with the combination of pembrolizumab and paclitaxel from 2016 to 2023.

Table 1 detailed the clinical characteristics of the patients, the majority of whom were women (n = 1112, 99.7%). In the Paclitaxel group, 99 patients (8.9%) weighed between 50 and 100 kg, compared to 63 patients (5.6%) in the Pembrolizumab group and 146 patients (12.4%) in the combined treatment group. Regarding age, 156 patients (14%) in the Paclitaxel group, 159 patients (14.2%) in the Pembrolizumab group, and 305 patients (27.3%) in the combined treatment group were under 64 years old. Mortality due to AEs was recorded in 22 patients (2%) in the combined treatment group, 12 patients (1.5%) in the Paclitaxel group, and 15 patients (1.1%) in the Pembrolizumab group. Detailed data was provided in Table 1. Before 2017, adverse reaction incidents were fewer than 10 cases annually. However, from 2018 to 2021, the incidence increased, with 13, 19, 15, and 19 cases respectively. In 2022 and 2023, the increased use of immunotherapy for breast cancer led to a rise in adverse reactions, reaching 146 and 330 cases respectively (Figure 2).

**TABLE 1:** Clinical characteristics of breast cancer patients in different groups.

Characteristics	Paclitaxel (n, %)	Pembrolizumab (n, %)	Combined treatment (n, %)
Sex			
Female	203(18.20)	360(32.30)	549(49.20)
Male	1(0.10)	2(0.20)	1(0.10)
Weight			
<50 kg	13(1.20)	9(0.80)	20(1.80)
>100 kg	7(0.60)	8(0.70)	14(1.30)
50∼100 kg	99(8.90)	63(5.60)	146(12.40)
Missing	85(7.60)	285(25.50)	370(31.40)
Age			
18∼64	156(14.00)	159(14.20)	305(27.30)
65∼85	38(3.40)	76(6.80)	93(8.30)
Missing	10(0.90)	127(11.40)	152(13.60)
Outcome			
Death	12(1.10)	15(1.50)	22(2.00)
Disability	5(0.40)	6(0.50)	9(0.80)
Hospitalization - Initial or Prolonged	83(7.40)	87(7.80)	166(14.90)
Life-Threatening	11(1.00)	8(0.70)	20(1.80)
Other Serious (Important Medical Event)	90(8.10)	230(20.60)	309(27.70)
Missing	3(0.30)	22(2.00)	24(2.20)

### Statistical analysis of adverse events in different treatment groups

A statistical analysis of adverse events was performed across various treatment groups based on SOC standards. The results indicate that general disorders and administration site conditions had the highest occurrence rate among overall adverse events (N = 2877, 13.30%) (Table 2). This was followed by respiratory, thoracic, and mediastinal disorders (N = 2032, 9.39%), gastrointestinal disorders (N = 1993, 9.21%), nervous system disorders (N = 1846, 8.53%), and skin and subcutaneous tissue disorders (N = 1735, 8.02%).

**TABLE 2:** System organ classes (SOCs) for adverse events of combined treatment, paclitaxel and pembrolizumab.

SOCs	Total (n, %)	Combined treatment (n, %)	Paclitaxel (n, %)	Pembrolizumab (n, %)
General disorders and administration site conditions	2877(13.3)	224(12.48)	2300(13.20)	353(14.67)
Respiratory, thoracic and mediastinal disorders	2032(9.39)	73(4.07)	1863(10.69)	96(3.99)
Gastrointestinal disorders	1993(9.21)	130(7.24)	1691(9.70)	172(7.15)
Nervous system disorders	1846(8.53)	94(5.24)	1609(9.23)	143(5.94)
Skin and subcutaneous tissue disorders	1735(8.02)	112(6.24)	1507(8.65)	116(4.82)
Blood and lymphatic system disorders	1297(6.00)	215(11.98)	896(5.14)	186(7.73)
Investigations	1224(5.66)	187(10.42)	854(4.90)	183(7.60)
Infections and infestations	1043(4.82)	96(5.35)	852(4.89)	95(3.95)
Neoplasms benign, malignant and unspecified	1003(4.64)	72(4.01)	774(4.44)	157(6.52)
Musculoskeletal and connective tissue disorders	928(4.29)	45(2.51)	783(4.49)	100(4.15)
Vascular disorders	892(4.12)	35(1.95)	837(4.80)	20(0.83)
Injury, poisoning and procedural complications	867(4.01)	101(5.63)	507(2.91)	259(10.76)
Cardiac disorders	724(3.34)	34(1.89)	642(3.68)	48(1.99)
Hepatobiliary disorders	616(2.84)	52(2.90)	467(2.68)	97(4.03)
Metabolism and nutrition disorders	498(2.30)	53(2.95)	376(2.16)	69(2.87)
Immune system disorders	415(1.91)	31(1.73)	367(2.11)	17(0.71)
Eye disorders	381(1.76)	10(0.56)	341(1.96)	30(1.25)
Renal and urinary disorders	326(1.51)	40(2.23)	246(1.41)	40(1.66)
Psychiatric disorders	311(1.44)	15(0.84)	254(1.46)	42(1.74)
Endocrine disorders	232(1.07)	95(5.29)	37(0.21)	100(4.15)
Surgical and medical procedures	151(0.70)	39(2.17)	47(0.27)	65(2.70)
Ear and labyrinth disorders	56(0.26)	2(0.11)	54(0.31)	0(0.00)
Reproductive system and breast disorders	56(0.26)	6(0.33)	45(0.26)	5(0.21)
Social circumstances	54(0.25)	25(1.39)	22(0.13)	7(0.29)
Congenital, familial and genetic disorders	52(0.24)	7(0.39)	42(0.24)	3(0.12)
Product issues	22(0.10)	2(0.11)	16(0.09)	4(0.17)

In the combined treatment group, general disorders and administration site conditions again showed the highest occurrence rate (N = 224, 12.48%), followed by blood and lymphatic system disorders (N = 215, 11.98%). Investigations (N = 187, 10.42%), gastrointestinal disorders (N = 130, 7.24%), and skin and subcutaneous tissue disorders (N = 112, 6.24%) also exhibited high occurrence rates.

For the pembrolizumab group, the highest incidence was in general disorders and administration site conditions (N = 353, 14.67%), followed by injury, poisoning, and procedural complications (N = 259, 10.76%). Blood and lymphatic system disorders (N = 186, 7.73%), investigations (N = 183, 7.60%), and gastrointestinal disorders (N = 172, 7.15%) also showed significant incidence rates.

In the paclitaxel pembrolizumab group, the highest incidence was observed in general disorders and administration site conditions (N = 2300, 13.20%), followed by respiratory, thoracic, and mediastinal disorders (N = 1863, 10.69%). Gastrointestinal disorders (N = 1691, 9.70%), nervous system disorders (N = 1609, 9.23%), and gastrointestinal disorders (N = 1507, 8.65%) also reported high incidence rates. Detailed information is presented in Table 2.

### Adverse event spectrum of paclitaxel combined with pembrolizumab treatment

**FIGURE 3:**
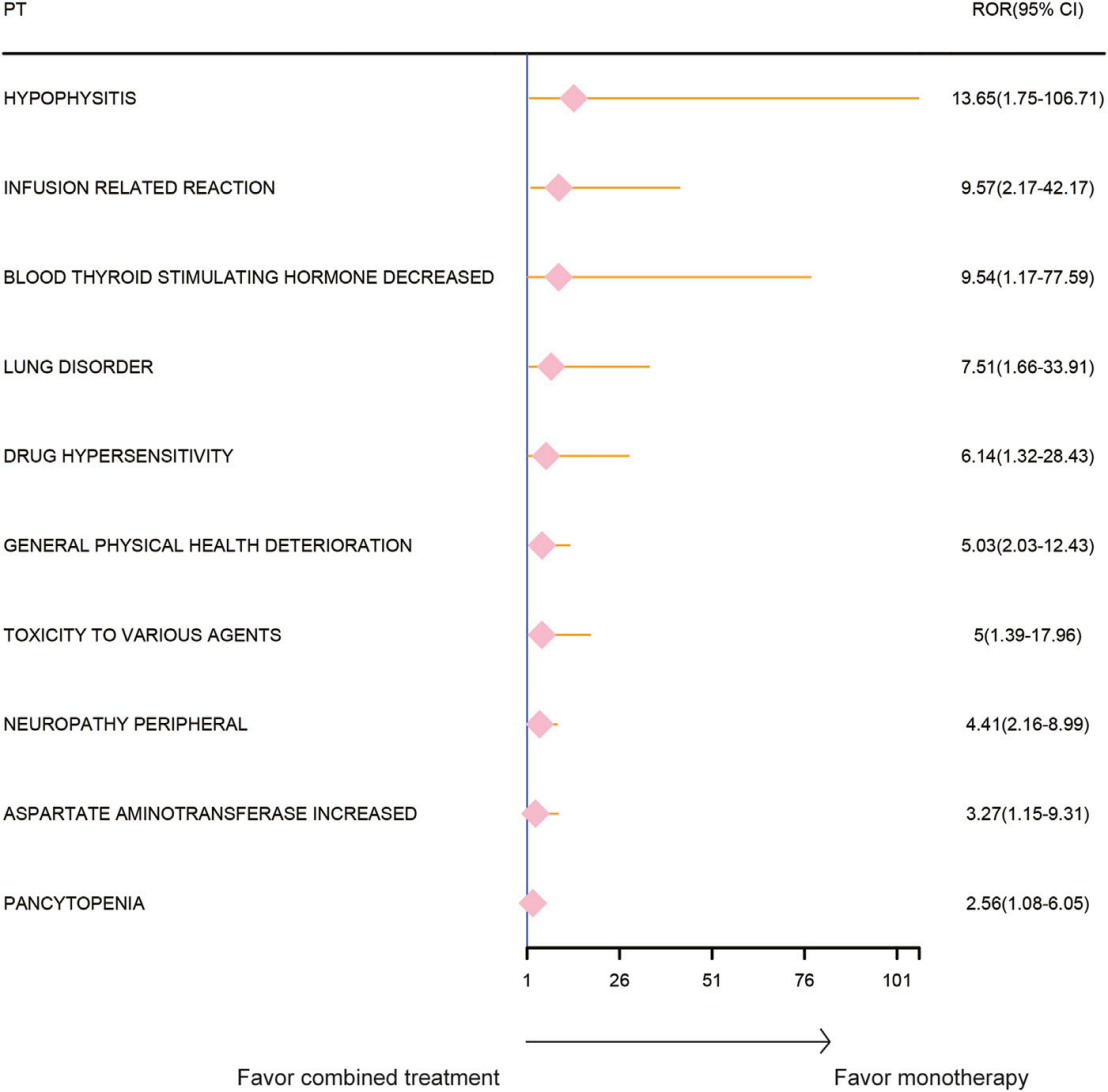
Safety signals (ROR) comparing pembrolizumab combined with paclitaxel to pembrolizumab monotherapy in breast cancer. This figure highlights the top 10 adverse event risks according to ROR. ROR, reporting odds ratios. Arrows to the right indicate a high risk of adverse events in the combination therapy group, supporting monotherapy.

**FIGURE 4:**
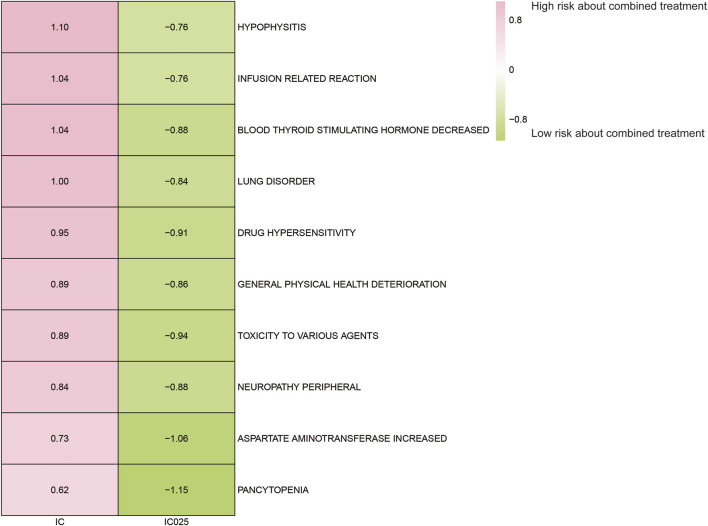
Safety signals (IC and IC025) comparing pembrolizumab combined with paclitaxel to pembrolizumab monotherapy in breast cancer. Top 10 adverse event risks according to ROR are displayed. ROR, reporting odds ratios; IC, information component; IC025, the lower limit of the 95% confidence interval of IC.

**FIGURE 5:**
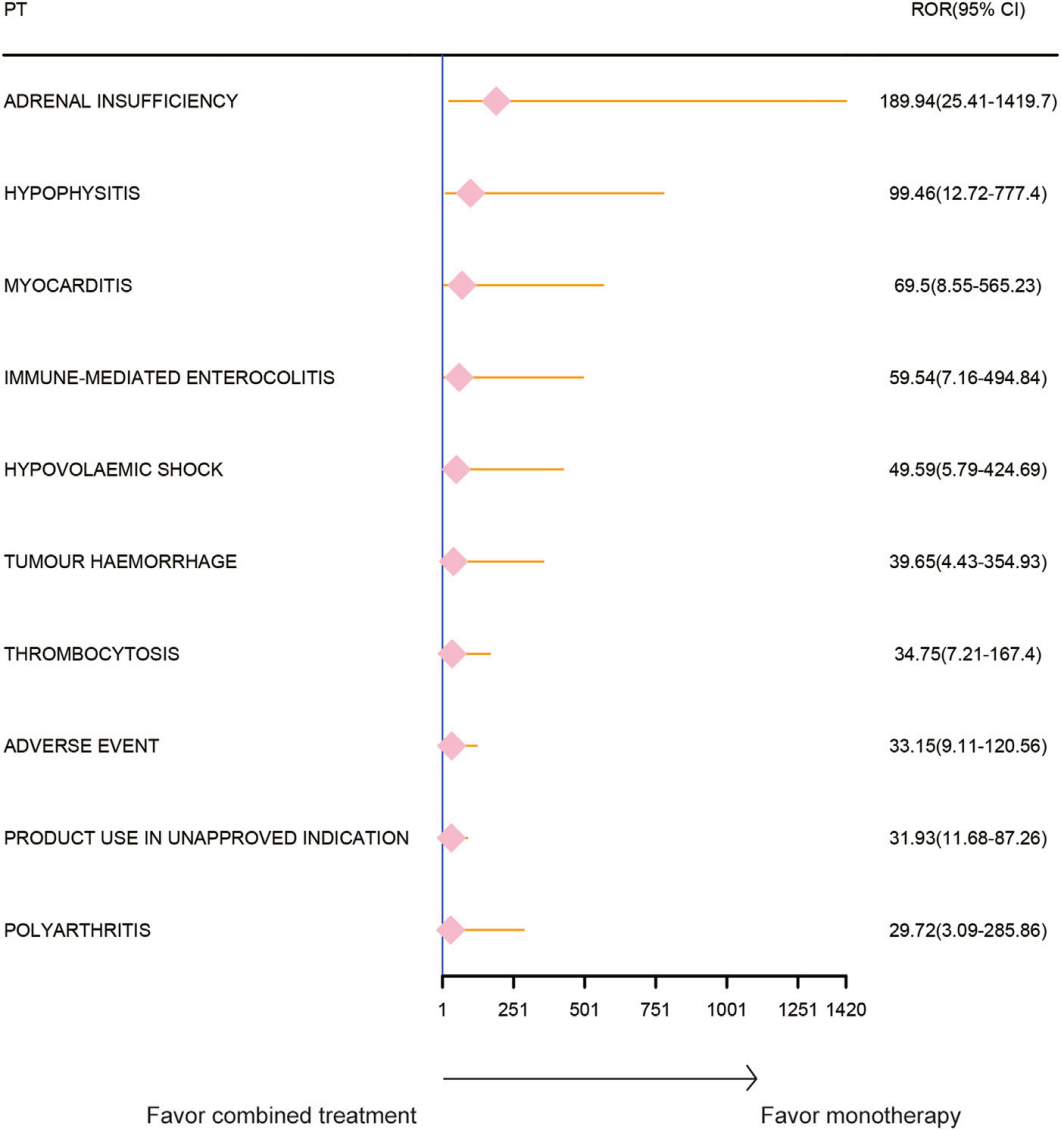
Safety signals (ROR) comparing pembrolizumab combined with paclitaxel to paclitaxel monotherapy in breast cancer. This figure highlights the top 10 adverse event risks according to ROR. ROR, reporting odds ratios. Arrows to the right indicate a high risk of adverse events in the combination therapy group, supporting monotherapy.

**FIGURE 6:**
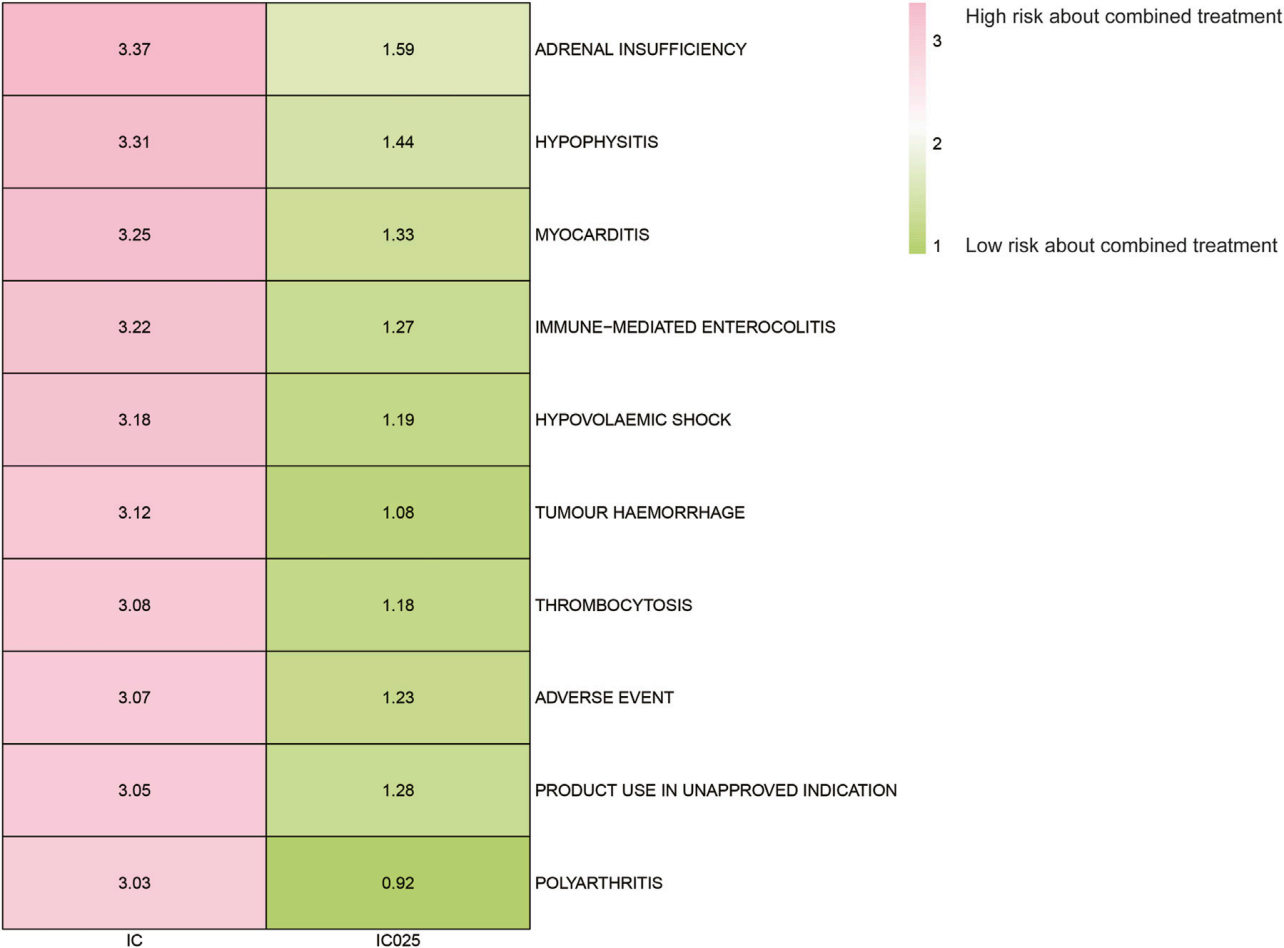
Safety signals (IC and IC025) comparing pembrolizumab combined with paclitaxel to paclitaxel monotherapy in breast cancer. The figure presents the top 10 adverse event risks according to ROR. ROR refers to reporting odds ratios, IC represents the information component, and IC025 denotes the lower limit of the 95% confidence interval of IC.

The AE spectrum resulting from paclitaxel and pembrolizumab combination therapy was presented in Figures 3–6, analyzed using the ROR and Bayesian confidence propagation neural network (BCPNN) algorithm. Analysis of ROR (Figure 3) and IC values (Figure 4) indicated no significant difference in AEs between the combination therapy and pembrolizumab-only groups. However, a marked increase in AE risk was noted in the combination therapy group compared to the paclitaxel-only group (Figures 5, 6). The three most significant AE risk elevations were adrenal insufficiency (ROR = 189.94, 95% CI 25.41–1419.7, IC = 3.37, IC025 = 1.59), hypophysitis (ROR = 99.46, 95% CI 12.72–777.4, IC = 3.31, IC025 = 1.44), and myocarditis (ROR = 69.5, 95% CI 8.55–565.23, IC = 3.25, IC025 = 1.33). These results suggested that combination therapy was significantly associated with an increased risk of specific AEs, including adrenal insufficiency, hypophysitis, and myocarditis, compared to paclitaxel monotherapy.

### Comparison of time-to-set in different groups

**FIGURE 7:**
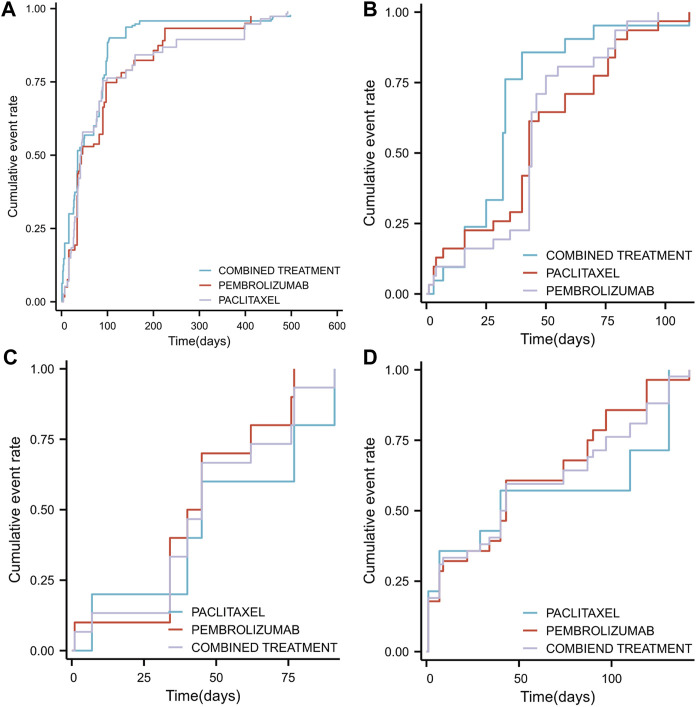
Timeline illustrating the duration from the initiation of pembrolizumab in combination with paclitaxel, pembrolizumab alone, or paclitaxel monotherapy to the occurrence of adverse events (SOC classification) **(A)** The median time to the occurrence of overall adverse events across different groups **(B)** The median time to the occurrence of adverse events related to general disorders and administration site conditions across different groups **(C)** The median time to the occurrence of adverse events related to respiratory, thoracic, and mediastinal disorders across different groups **(D)** The median time to the occurrence of adverse events related to gastrointestinal disorders across different groups.

The Time-to-Set of drug adverse events was analyzed across various groups (Figure 7A). Pembrolizumab monotherapy exhibited a median Time-to-Set of 43 (35–90) days, while paclitaxel monotherapy showed 42 (37–76) days. In contrast, combination therapy demonstrated a significantly shorter median Time-to-Set of 35 (34–70) days compared to both monotherapies. Additionally, the paclitaxel monotherapy group (HR 0.788, 95% CI 0.623–0.997; *p* = 0.046) and the pembrolizumab monotherapy group (HR 0.761, 95% CI 0.604–0.959; *p* = 0.021) exhibited a lower risk of adverse event incidence relative to the combination therapy group.

An analysis based on SOC classification criteria for adverse drug reaction events identified the top three adverse drug reaction events and examined variations in their median cumulative occurrence times across different treatment groups. The leading adverse events were: general disorders and administration site conditions, respiratory, thoracic and mediastinal disorders, and gastrointestinal disorders. The Time-to-Set for general disorders and administration site conditions was 32 days (25–40) for combination therapy, which was significantly shorter than for pembrolizumab monotherapy (44 days, 43–50, *p* < 0.05) and paclitaxel monotherapy (43 days, 40–70, *p* < 0.05) (Figure 7B). For respiratory, thoracic and mediastinal disorders, no significant difference was observed (*p* = 0.527) between combination therapy (45 days, 34–77) and either pembrolizumab (42.5 days, 40-Inf) or paclitaxel monotherapy (42.5 days, 40-Inf) (Figure 7C). Similarly, for gastrointestinal disorders, the differences were not significant (*p* = 0.726) between combination therapy (41.5 days, 29–87), pembrolizumab (43 days, 22–87), and paclitaxel monotherapy (40 days, 7-Inf) (Figure 7D).

**FIGURE 8:**
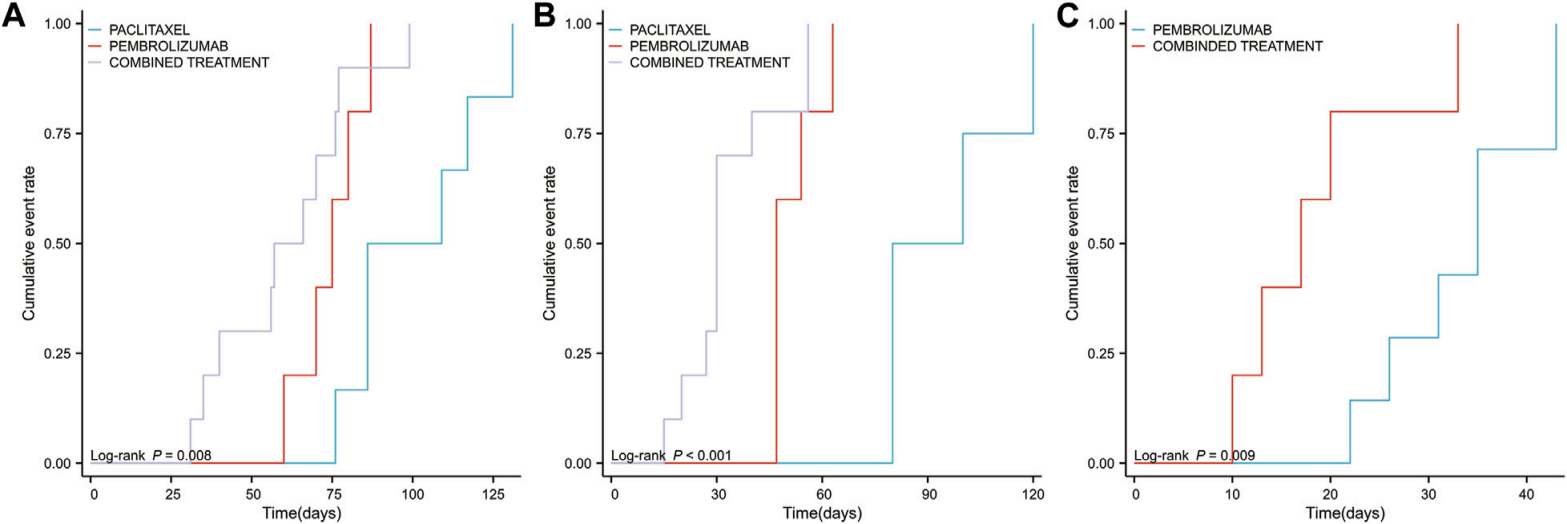
Timeline illustrating the duration from the initiation of pembrolizumab in combination with paclitaxel, pembrolizumab alone, or paclitaxel monotherapy to the occurrence of the top three high-risk PTs (available for statistical analysis) **(A)** The median time to the occurrence of adverse events related to adrenal insufficiency across different groups **(B)** The median time to the occurrence of adverse events related to myocarditis across different groups **(C)** The median time to the occurrence of adverse events related to immune-related enterocolitis across different groups.

In addition, an analysis was conducted on the three PTs exhibiting the most significant differences between combination therapy and monotherapy, focusing on variations in cumulative occurrence time and cumulative incidence rate across different groups. The results indicated that the combination therapy group experienced significantly shorter intervals to the onset of adrenal insufficiency (*p* = 0.008, Figure 8A), myocarditis (*p* < 0.001, Figure 8B), and immune-related enterocolitis (*p* = 0.009, Figure 8C) compared to the monotherapy group.

## Discussion

This study rigorously evaluated the variance in AE risk between combined pembrolizumab and paclitaxel treatment *versus* monotherapy with either agent. The data suggest that combination therapy may be linked to an increased incidence of adverse events, potentially impacting patients’ quality of life.

The significant difference in adverse event incidence between the paclitaxel monotherapy group and the combination therapy group is primarily attributed to immunotherapy drug side effects. Immunotherapy-related AEs are influenced by various factors, with age being a critical determinant. The incidence and nature of immunotherapy-related AEs vary by age group. A study indicated that among patients aged 65 to 85 receiving pembrolizumab, the most common AEs were cardiac, renal, urinary disorders, and metabolic and nutritional disorders. In contrast, patients under 65 experienced reproductive system, hepatic, and hematologic disorders more frequently (Yang et al., 2023). This phenomenon is closely linked to age-related changes in immune system function (Huang et al., 2021; Wong et al., 2021). As individuals age, immune system functionality diminishes, characterized by dysregulation, including heightened autoimmunity and reduced defense against infections and cancer, a process known as “immune aging” (Yang et al., 2023). These alterations can compromise the safety and efficacy of immune-based therapies, potentially increasing cancer and respiratory disease incidences (Gubbels Bupp et al., 2018; Ye et al., 2020; Kang et al., 2021). The relationship between elderly patients and immunotherapy-induced AEs remains a subject of ongoing debate. A study indicated that elderly patients had a higher incidence of pulmonary toxicity during ICI (anti-PD-1/L1) treatment (Huang et al., 2021), while another study demonstrated that elderly patients showed better tolerance to ICI therapies (Pawelec, 2019; Nebhan et al., 2021). Extensive missing age-related information in both the immunotherapy and combination therapy groups precluded further stratified analysis by age in this study. Regarding the types of adverse events observed, regimens including pembrolizumab were more likely to cause immune-related adverse events involving the kidneys and heart compared to paclitaxel monotherapy, consistent with previous data (Yang et al., 2023). Further research is required to fully elucidate the impact of immunotherapy on adverse events in the elderly population.

Significant differences in the incidence of adverse events were observed between the paclitaxel monotherapy group and the combination therapy group, primarily due to immunotherapy drug side effects. However, no significant difference was found in the incidence of adverse events between the combination therapy group and the pembrolizumab monotherapy group. A retrospective study demonstrated that the incidence of adverse events in the combination therapy group did not significantly differ from that in the immunotherapy monotherapy group (Wang et al., 2021). Another study highlighted age differences between patients receiving monotherapy immunotherapy and those receiving combination therapy; the former group tended to be older (Pérol et al., 2022). Age affects the incidence of chemotherapy-related adverse events and tolerance to chemotherapy side effects. Consequently, older individuals are often prescribed monotherapy immunotherapy to mitigate chemotherapy side effects, while younger individuals, who generally tolerate these side effects better, are more likely to receive combination therapy. Implementing personalized treatment plans tailored to different age groups can minimize the difference in adverse event incidence between monotherapy immunotherapy and combination therapy recipients. Additionally, paclitaxel can be categorized into variants such as paclitaxel, docetaxel, and nab-paclitaxel. These formulations vary in solvents, carriers, and structures, affecting the incidence of chemotherapy-related adverse events (Dranitsaris et al., 2016). A randomized controlled study involving 1052 patients demonstrated that nab-paclitaxel, compared to paclitaxel, significantly reduces the incidence of grade ≥3 neuropathy, neutropenia, joint pain, and myalgia (Socinski et al., 2012). Utilizing paclitaxel formulations with fewer side effects, such as albumin-bound paclitaxel, mitigates the incidence of chemotherapy-related adverse reactions, thus narrowing the gap in adverse event occurrence rates relative to combination therapy.

An analysis was conducted on the time-to-set for receiving pembrolizumab monotherapy or paclitaxel monotherapy. The time-to-set for pembrolizumab monotherapy was 43 (35–90) days, and for paclitaxel monotherapy, it was 42 (37–76) days. In contrast, combination therapy had a significantly shorter time-to-set of 35 (34–70) days. Compared to the combination therapy group, both the paclitaxel monotherapy group (HR = 0.788, 95% CI = 0.623–0.997; *p* = 0.046) and the pembrolizumab monotherapy group (HR = 0.761, 95% CI = 0.604–0.959; *p* = 0.021) exhibited a lower risk of adverse event incidence. These results suggested that combination therapy significantly increased the incidence of adverse events. Additionally, the top three adverse events (general disorders and administration site conditions, respiratory, thoracic and mediastinal disorders, and gastrointestinal disorders) were analyzed for overall occurrence. Differences in median cumulative occurrence time of these adverse events among the different groups were also examined. For general disorders and administration site conditions, the time-to-set for combination therapy (32, 25–40 days) was shorter than both pembrolizumab monotherapy (44, 43–50 days) and paclitaxel monotherapy (43, 40–70 days). Additionally, the interval between the onset of adrenal insufficiency, myocarditis, and immune-related enterocolitis was significantly shorter in the combination therapy group compared to the monotherapy group. A meta-analysis reported an overall incidence of FAEs with pembrolizumab at 1.2% (95% CI: 0.5–2.8) (Sher et al., 2020). Compared to chemotherapy, the overall RR for FAEs in patients treated with pembrolizumab was 1.24 (95% CI: 0.8–1.9; *p* = 0.31) (Sher et al., 2020). The risk of FAEs with pembrolizumab monotherapy was similar to conventional chemotherapy (*p* = 0.35), whereas combining pembrolizumab with chemotherapy increased the risk of treatment-related mortality by 58%.

The combination therapy group exhibited a higher mortality rate compared to the monotherapy group, though the difference between the monotherapy groups was not significant. Further analysis revealed a significantly elevated risk of adrenal insufficiency, pituitary inflammation, myocarditis, and immune-related enterocolitis in the combination therapy group. Notably, adrenal insufficiency, myocarditis, and immune-related enterocolitis had significantly shorter onset intervals. This suggests an increased incidence of severe adverse events in the combination therapy group compared to the monotherapy group. Adrenal insufficiency, defined by cortisol deficiency, poses a life-threatening risk and can manifest as primary (adrenal), secondary (pituitary), or stage III (primarily adrenal suppression due to glucocorticoids or opioids), or as a consequence of immunotherapy medications (Macfarlane et al., 2020). Its prevalence is rising due to increased use of glucocorticoids, opioids, and immunotherapy drugs (Chalitsios et al., 2020). Evidence indicates that approximately 1%–2% of patients treated with PD-1/PD-L1 inhibitors experience adrenocorticotropic hormone (ACTH) deficiency (Morganstein and Larkin, 2020). A multicenter study observed adrenal insufficiency in 8 out of 54 patients undergoing AEs to immunotherapy (Goodman et al., 2023). Analysis revealed an elevated risk of adrenal insufficiency in the combined treatment group compared to paclitaxel monotherapy. This increased risk may be attributed to pembrolizumab, an immunotherapeutic agent, which can cause adrenal damage during anti-tumor therapy, resulting in adrenal insufficiency. Hypophysitis, associated with viral infections, immune disorders, heredity, and drug use (Joshi et al., 2018), is traditionally considered rare, with an annual incidence of 1 in 9 million (Joshi et al., 2018). However, the widespread use of immunotherapy has led to a rising incidence of hypophysitis (Baxi et al., 2018). The mechanisms linking hypophysitis to immunotherapy are twofold. Firstly, the pituitary gland shares targets with tumor antigens, eliciting similar reactivity. Immunotherapy drugs promote T cells to target tumors, inadvertently causing damage to the pituitary gland (Deligiorgi et al., 2021). Secondly, elevated interferons (IFN), particularly IFNγ, induce chemokine production (CXCL9 and CXCL10), stimulating chemotactic effects on T cells, prompting them to damage the pituitary gland (Chan and Bass, 2020). The study identified a significantly increased risk of hypophysitis in the combined therapy group compared to paclitaxel monotherapy, aligning with findings from previous breast cancer clinical studies (Shitara et al., 2018; Adams et al., 2019a; Winer et al., 2021). Myocarditis, an inflammatory heart disease, is characterized by diverse clinical manifestations and outcomes (Harris et al., 2021). Some patients may exhibit transient symptoms that resolve rapidly, while others experience severe complications such as cardiogenic shock or fatal arrhythmias (Bhatia et al., 2023). Studies examining the relationship between myocarditis and immunotherapy highlight the pivotal role of the thymus in T cell maturation, with thymic diseases amplifying the incidence and severity of immunotherapy-associated myocarditis and muscular toxicity (Fenioux et al., 2023). Chronic bidirectional ventricular tachycardia has been linked to immune checkpoint inhibitor myocarditis, underscoring its role in causing myocarditis (Zhao et al., 2023). The investigation revealed a significantly increased risk of myocarditis in the combination treatment group compared to paclitaxel monotherapy. This heightened risk is likely due to use of pembrolizumab, an immunotherapy drug that elevates the risk of immune-associated myocarditis while improving patient prognosis.

Previous studies have demonstrated that drug interactions, including synergistic and antagonistic effects, can lead to drug-drug interactions (DDI), resulting in adverse events (Alnaim et al., 2022). The efficacy of combination therapy, compared to monotherapy, reflects a complex relationship that goes beyond the simple arithmetic sum of 1 + 1 = 2. Similarly, the occurrence of adverse reactions is not merely additive. Approximately 60% of cancer patients experience DDI events due to the concurrent use of two or more drugs. The number of medications is a significant risk factor for DDI events, with treatment type (e.g., chemotherapy) and hospitalization duration also showing notable associations (Alnaim et al., 2022). Pharmacological studies reveal a high prevalence of underlying DDI, with an overall event rate of 78%, and most patients experiencing 1-2 potential DDI occurrences (39.2%). Adverse drug events include various outcomes such as reduced treatment response, prolonged QT interval, tendon rupture, myelosuppression, and neurotoxicity (Ismail et al., 2020). A comparative analysis of different treatment regimens indicated significant differences in the risk of specific adverse events, suggesting that these differences may be attributed to drug interactions.

Choosing between combination therapy and monotherapy necessitates considering factors such as tumor characteristics, adverse events, and patients’ physical condition to develop individualized treatment regimens. For patients with PD-L1 expression exceeding 50%, monotherapy immunotherapy achieves comparable efficacy to combination therapy and reduces adverse reactions (Brahmer et al., 2017). Conversely, for those with PD-L1 expression below 50%, monotherapy immunotherapy is less effective, necessitating combination therapy for improved prognosis (Paz-Ares et al., 2020). A high tumor burden and elevated NLR indicate poor response to monotherapy immunotherapy, thus requiring combination therapy despite potential increased side effects (Hwang et al., 2022). Additionally, treatment plans should consider patient lifestyle and basic characteristics. Older patients may have reduced tolerance to combination therapy, increasing the risk of cardiac toxicity and hematologic adverse events, thus favoring monotherapy (Li et al., 2020). Non-smoking patients generally benefit more from combination therapy compared to smokers (Pérol et al., 2022). Although chemotherapy combined with immunotherapy improves prognosis, it also increases the incidence of adverse events such as proteinuria, anemia, leukopenia, neutropenia, and thrombocytopenia (Li et al., 2020). In summary, balancing adverse reactions and treatment efficacy through personalized monotherapy and combination strategies can enhance patient quality of life and prognosis.

Despite the valuable insights gained, the study has limitations. The small number of cases receiving combination therapy in the database may influence the results, necessitating further validation through additional studies. Moreover, the FAERS database primarily reflects European and American populations, with limited representation from Asia and other regions, introducing regional limitations. Additionally, insufficient demographic and disease biology data in the database impede further analysis to determine the disease incidence rate and its potential impact mechanisms. Furthermore, some spontaneous reports may be biased. A more comprehensive exploration of AE risks in combination regimens across diverse geographical areas is recommended.

## Conclusion

Analysis of the FAERS database revealed that combination therapy significantly elevates the risk of adrenal insufficiency, myocarditis, hypophysitis, and immune-related enterocolitis compared to paclitaxel monotherapy. These findings provide a valuable reference for clinicians in anticipating and managing potential AEs associated with this treatment regimen.

## Data Availability

The original contributions presented in the study are included in the article/supplementary material, further inquiries can be directed to the corresponding author.
